# Up-regulation of MTHFD2 is associated with clinicopathological characteristics and poor survival in ovarian cancer, possibly by regulating MOB1A signaling

**DOI:** 10.1186/s13048-022-00954-w

**Published:** 2022-02-08

**Authors:** Xiangrong Cui, Huancheng Su, Jiaolin Yang, Xueqing Wu, Kai Huo, Xuan Jing, Sanyuan Zhang

**Affiliations:** 1 Reproductive Medicine Center, The affiliated Children’s Hospital of Shanxi Medical University, Children’s Hospital of Shanxi, Women Health Center of Shanxi, Taiyuan, 030001 China; 2 Gynaecology and Obstetrics Department, First Hospital of Shanxi Medical University, Taiyuan, 030001 China; 3 Clinical Laboratory, The Affiliated People’s Hospital of Shanxi Medical University, Taiyuan, 030001 China; 4 Breast Surgery Department, Tumor Hospital of Shanxi, Affiliated of Shanxi Medical University, Taiyuan, 030000 China

**Keywords:** MTHFD2, Ovarian cancer, Prognosis, MOB1A

## Abstract

**Background:**

MTHFD2 is a folate-coupled metabolic enzyme, which has been proved to participant in the metabolic reprogramming and tumor cell-sustaining proliferative capacity. However, the function of MTHFD2 in the development of ovarian cancer and its potential molecular mechanisms is still unclear.

**Materials and methods:**

The expression, various mutations, prognosis, and related network signaling pathways of MTHFD2 were analyzed using bioinformatics-related websites, including Oncomine, GEPIA, UCSC, cBioPortal, KM Plotter, TISIDB and TIMER. The prognostic value of MTHFD2 expression was validated by our own ovarian cancer samples using RT-qPCR. The migration ad invasion of ovarian cancer cells were further analyzed by CCK-8 and transwell assay. The Western-blot assay was performed to explore the protein levels of MTHFD2 and MOB1A.

**Results:**

We obtained the following important results. (1) MTHFD2 expression was markedly up-regulated in ovarian cancer than normal samples. (2) Among patients with ovarian cancer, those with higher MTHFD2 expression was associated with lower survival rate. (3) The major mutation type of MTHFD2 in ovarian cancer samples was missense mutation. (4) MTHFD2 knockdown inhibited proliferation, migration, invasion, as well as the expression of MOB1A in vitro.

**Conclusion:**

MTHFD2, as a NAD + -dependent enzyme, accelerated tumor progression by up-regulating MBO1A, suggesting that this protein may be an independent prognostic factor and a potential therapeutic target for future ovarian cancer treatments.

**Supplementary Information:**

The online version contains supplementary material available at 10.1186/s13048-022-00954-w.

## Introduction

Ovarian cancer (OC) is recognized as the most common and lethal gynecologic malignancies, which characterized by multidrug resistance, early-onset metastasis, recurrence, and poor prognosis [9, 11, 14]. Due to a wide range of clinical, histopathological, and molecular heterogeneity, most patients were identified in the advanced stage at the time of initial diagnosis, causing an unsatisfactory 5-year survival rate of ovarian cancer patients [11, 27]. Therefore, it is urgent to screen and identify reliable biomarkers to realize the accuracy of early diagnosis for ovarian cancer. Besides, understanding the pathophysiological mechanism of ovarian cancer may promote the optimization of treatment plan.

MTHFD2 (the mitochondrial methylenetetrahydrofolate dehydrogenase/cyclohydrolase), a folate-coupled metabolic enzyme, expressed in the developing embryo and is absent in most adult tissues [1, 10]. Interestingly, recent studies have demonstrated that MTHFD2 confers redox homeostasis and drives cancer cell proliferation and migration [16, 20]. Markedly elevated expression of MTHFD2 was identified in many cancers and correlates with poor survival in breast cancer patients [13, 16]. Furthermore, repression of MTHFD2 decreased the invasion and migration of breast cancer cell lines [7]. However, the exact role of MTHFD2 in ovarian cancer and its underlying regulatory mechanism have not been elucidated.

Therefore, the current study devoted to exploring the potential value of MTHFD2 for the diagnostic and prognostic of ovarian cancer, as well as its underlying regulation mechanism. We adopt bioinformatics methods to observe the expression ad prognostic value of MTHFD2 in ovarian cancer. Besides, we used ovarian cancer cell lines to analyze the regulatory mechanism underlying the effect of highly expressed MTHFD2 on ovarian cancer. Our results demonstrated that MTHFD2 expression in ovarian cancer was up-regulated and associated with poor prognosis in ovarian cancer patients, which may play a role in malignant transformation mainly through MOB1A signaling pathway. These observations will help to develop and optimize new insights into the diagnosis and prognosis of ovarian cancer.

## Materials and methods

### Human sample and clinicopathological data collection

This study was received the approval of the Ethics Committee of Shanxi Medical University (Ethical code: 2019220221). Frozen tissues specimens were collected 35 patients with ovarian cancer without any therapy before surgery at the Department of Gynaecology and Obstetrics from Tumor Hospital of Shanxi (China). The average age of the patients was 53.9 years (24-69 years). In formed written consent was obtained from all patients enrolled.

### Cell culture and transfections

This human ovarian cancer cell line SKOV3 was purchased from the American Type Culture Collection (ATCC) and cells were cultured in RPMI medium 1640 (Gibco, Thermo Fisher Scientifc, Inc.) supplemented with 10% fetal bovine serum (FBS; HyClone, Logan, USA) and 1% penicillin/streptomycin (Invitrogen, Waltham, MA) in a stable humidified atmosphere with 5% CO2 at 37 °C. siRNA targeting MTHFD2 (si-MTHFD2) and siRNA negative control (si-NC) were constructed and purchased from GenePharma (Shanghai, China).

### Oncomine database verification

The Oncomine (www.oncomine.org) platform is a public bioinformatics network database including 715 independent datasets and 86, 733 samples which has become an industry-standard tool cited in more than 1100 peer-reviewed journal articles [22]. MTHFD2 gene expression levels in malignancies were analyzed, selecting the cancer type as ovarian cancer. Paired Student’s t-test was performed to compare group means. A fold-change of at least 2 with a *P*-value < 0.001 was used as clinically significant, as previously described.

### Gene expression profiling interactive analysis

Gene Expression Profiling Interactive Analysis (GEPIA) database (http://gepia.cancer-pku.cn/) was performed to explore the data of tumor/normal differential expression analysis, profiling according to cancer types or pathological stages, patient survival analysis, similar gene detection, correlation analysis and dimensionality reduction analysis from TCGA and GTEx databases with bioinformatics tools CIBERSORT, EPIC, and quan Tlseq [26]. In GEPIA, the expression of MTHFD2 and MOB1A in various human cancers and adjacent normal tissues was obtained, furthermore the expression of MTHFD2 in ovarian cancer and corresponding normal tissues was analyzed.

### University of California Santa Cruz (UCSC) Cancer genomics browser analysis

UCSC Xena platform (http://xenabrowseer.net), included 758 cases of ovarian cancer with genomic and clinical, was utilized to access TCGA ovarian cancer data [6]. The relationship between the mRNA expression of MTHFD2 and MOB1A was conducted thorugh TCGA-Ovarian Cancer.

### cBioportal database analysis

cBioPortal (http://chioportal.org) was utilized to analyze MTHFD2 alterations observed in ovarian serous cystadenocarcinoma (TCGA, Nature 2011, *n* = 489) [2, 5]. By analyzing various types of mutations, putative copy number variations, and co-expression data, the tab OncoPrint displayed an overview of the genetic changes in each sample as gene mutations and heat maps of MTHFD2 expression.

### UALCAN analysis

UALCAN (http://ualcan.path.uab.edu), an an open public web resource, is utilized to analyze the mRNA expression of potential genes in various tumor subtypes, including age, gender, tumor stages, and other clinicopathological features [3]. In our study, UALCAN was pertormed to access the MTHFD2 expression levels in primary ovarian carcinoma tissues and its association with multiple clinical and pathological parameters.

### LinkedOmics analysis

The LinkFinder module of LinkedOmics (http://www.linkedomics.org/login.php), included multi-omics data from all 32 TCGA Cancer types and 10 Clinical Proteomics Tumor Analysis Consortium (CPTAC) cacner cohorts, is utilized to search for attributes that are associated with a query attribute, such as mRNA or protein expression signatures of genomic alterations, candidate biomarkers of clinical attributes, and candidate target genes of transcriptional factors, microRNAs, or protein kinases [28]. In our study, LinkFinder module was performed to identify differentially expressed genes related to MTHFD2 in the TCGA OV section. To derive biological insights from the association results, the LinkInterpreter module performs enrichment analysis based on Gene Ontology, biological pathways, network modules, among other functional categories.

### Kaplan Meier plotter

The Kaplan Meier plotter (http://kmplot.com/analysis/) is capable to evaluate the role of 2190 genes on survival in ovarian cancer from the databases containing GEO, EGA, and TCGA [15]. We accessed Kaplan Meier plotter to analyze the prognostic value of MTHFD2 in ovarian cancer. The patient samples were split into two groups by median expression to analyze the overall survival (OS) with hazard ratios (HRs) with 95% confidence intervals and log-rank *p*-values.

### TIMER database analysis

Tumor Immune Estimation Resource (TIMER, https://cistrome.shinyapps.io/timer/) is a website for comprehensive analysis of the tumor-infiltrating immune cells and gene expression in different types of cancers [12]. We evaluated the expression of MTHFD2 in ovarian cancer in relation to tumor purity and the abundance of immune infiltrating cells including B cells, CD8^+^ T cells, CD4^+^ T cells, macrophages, neutrophils, and dendritic cells. The levels of gene expression were expressed as Log2 RSEM.

### TISIDB analysis

TISIDB database (http://cis.Hku.hk/TISIDB) is a websit for comprehensive analyzing tumor and immune cell interactions which integrates into various heterogeneous data types for each gene [23]. We access this platform to analyze the relationship between MTHDF2 expression and tumor-infiltrating lymphocytes.

### Quantitative RT-PCR (qRT-PCR)

Total RNA of tissues and cells was extracted with TRIzol reagent (Invtrogen, Carlsbad, CA, USA) and the concentration of total RNA was measured with Nano-DropTM Spectrophotometers (Thermo). Appropriate amounts of total RNA were reverse transcribed using the Prime Script™ RT Master Mix kit (Thermo Fisher Scientic, Carlsbad, CA, USA). Real-Time PCR was performed with the SYBR® Premix Ex Taq Kit (TaKaRa, Otsu, Shiga, Japan) and primers (listed below) using CFX96 (Bio-Rad company, Shanghai, China). The following PCR primer sequences as follows: MTHFD2, 5′-GATCCTGGTTGGCGAGAATCC-3′ (forward) and 5′-TCTGGAAGAGG CAACTGAACA-3′ (reverse), MOB1A, 5′-CAGCAGCCGCTCTTCTAAAAC-3′ (forward) and 5′-CCTCAGGCAACATAACAGCTTG-3′ (reverse), GAPDH, 5′-ACCACAGTCCATGCCATCA C-3′ (forward); and 5′-TCCACCACCCTGTTGCTGTA-3′ (reverse). The method of 2^−ΔΔCt^ was performed to calculate the relative mRNA levels of target genes.

### Western blot

Total protein from ovarian carcinoma cell lines was extracted using lysis buffer (KeyGEN BioTECH, China) containing phosphatase and protease inhibitors (KeyGEN BioTECH, China). Protein concentration was detected using BCA protein assay kit (KeyGEN BioTECH, China). Equal amount of protein (10 μg) was loaded onto 10% SDS-PAGE (PG112, Epizyme Biotech, China) and then transferred to PVDF membranes (Millipore, USA). All membranes were then blocked in TBST for 1 h with 5% BSA at room temperature and followed by primary antibodies at 4 °C overnight. Then membranes were incubated at room temperature for 2 h with HRP-conjugated secondary antibodies (701,051, Zen Bioscience, China; ZB-2301, ZSGB-BIO, China). Protein signals were visualized with a chemiluminescence kit (Millipore, USA) and an Image system (Bio-Rad, USA). GAPDH was used as the internal control. Primary antibodies were as follows: MTHFD2 (ab151447, 1:3000, Abcam, Cambridge, MA, USA), MOB1A (ab236969, 1:3000, Abcam, Cambridge, MA, USA) and GAPDH (cat.no. T0004; 1:5000; Affinity Biosciences).

### CCK-8 detection of viability

Cell Counting Kit-8 was accessed to evaluate the cell viability. Cells were seeded into 96-well plates at 5 × 10^3^ cells per well and subjected in 100 μl serum-free medium for 24, 48, 72, and 96 h. Then, 10 μl CCK-8 solution (CCK-8, ATgene, Taiwan, China) was added to each well for 2 h and the absorbance (OD) values was detected at 450 nm by microplate reader (BioTek, Epoch, VT). All tests were repeated eight times, and each experiment was experimented at least three replicate.

### Cell migration and invasion ability assay

Invasion and migration abilities of ovarian cancer cells transfected with si-MTHFD2 and si-NC were determined using transwell migration and invasion assay. 600 μl DEME/F12 supplemented with 10% FBS was added to lower chambers, followed by Transwell chamber (Millipore, Burlington, MA, USA) seeded with 5 × 10^4^ cells. For cell invasion assay, 100 μg/ml Matrigel (BD Biosciences, Boston, MA, USA) was added to the upper layer of chamber. After incubating for 24 h, the cells in the upper chamber were removed. Cells were fixed with 600 μl methanol for 10 min. The chamber was stained with 0.25% cfystal violet (Solarbio) for 15 min. Finally, an inverted microscope (Eclipse Ti2; Nikon Corporation) was performed to photograph and calculate the invading and migratory cells in three random fields.

### Statistical analysis

GraphPad Prism 7.0 software was used to statistical analysis. Student’s t-test was performed to compare the differences between two groups. One-way ANOVA was performed to compare multiple groups. Overall survival was presented as Kaplan-Meier survival curves and the statistical comparisons were calculated by Log-rank test. *P*-value < 0.05 was set as the threshold.

## Results

### MTHFD2 is up-regulated in human ovarian cancer

**Fig. 1:**
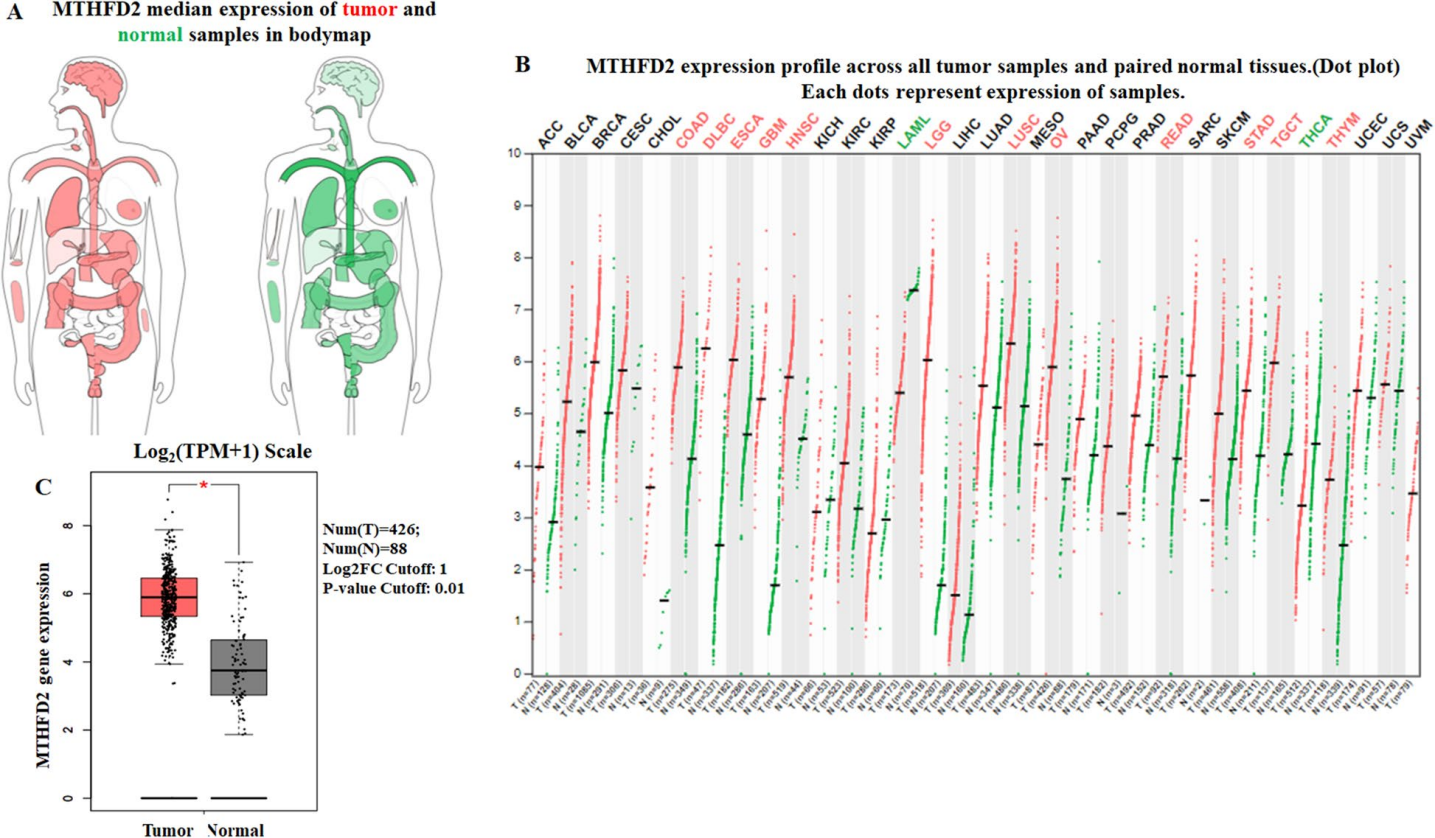
Expression of MTHFD2 in ovarian cancer and normal tissues from GEPIA databases. **A** MTHFD2 median expression of tumor (red) and normal (green) samples in body map. **B** MTHFD2 expression profile across all tumor (red) and paired normal (green) tissues. Each dot represents the expression of sample. **C** The expression of MTHFD2 mRNA in ovarian cancer tissues (red box) and paired normal tissues (black box) from GEPIA

**Fig. 2:**
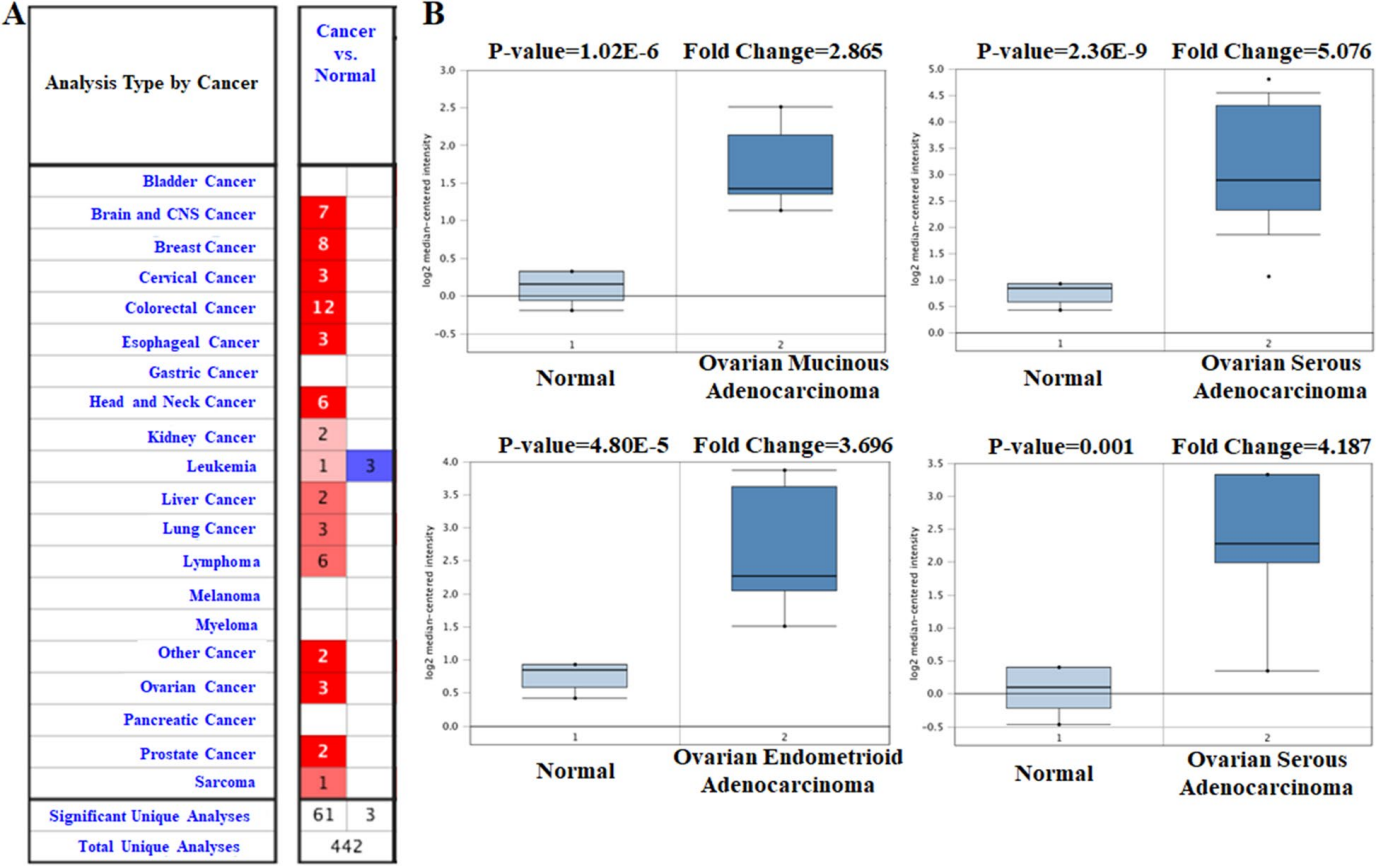
MTHFD2 analysis in ovarian cancer (Oncomine database). **A** Expression of MTHFD2 in different tumors. Graphs show the number of datasets with statistically significant mRNA overexpression (red) or down-regulation (blue) of MTHFD2. **B** Box plots derived from gene expression data in Oncomine comparing the expression of MTHFD2 in normal and ovarian cancer tissue

**Fig. 3:**
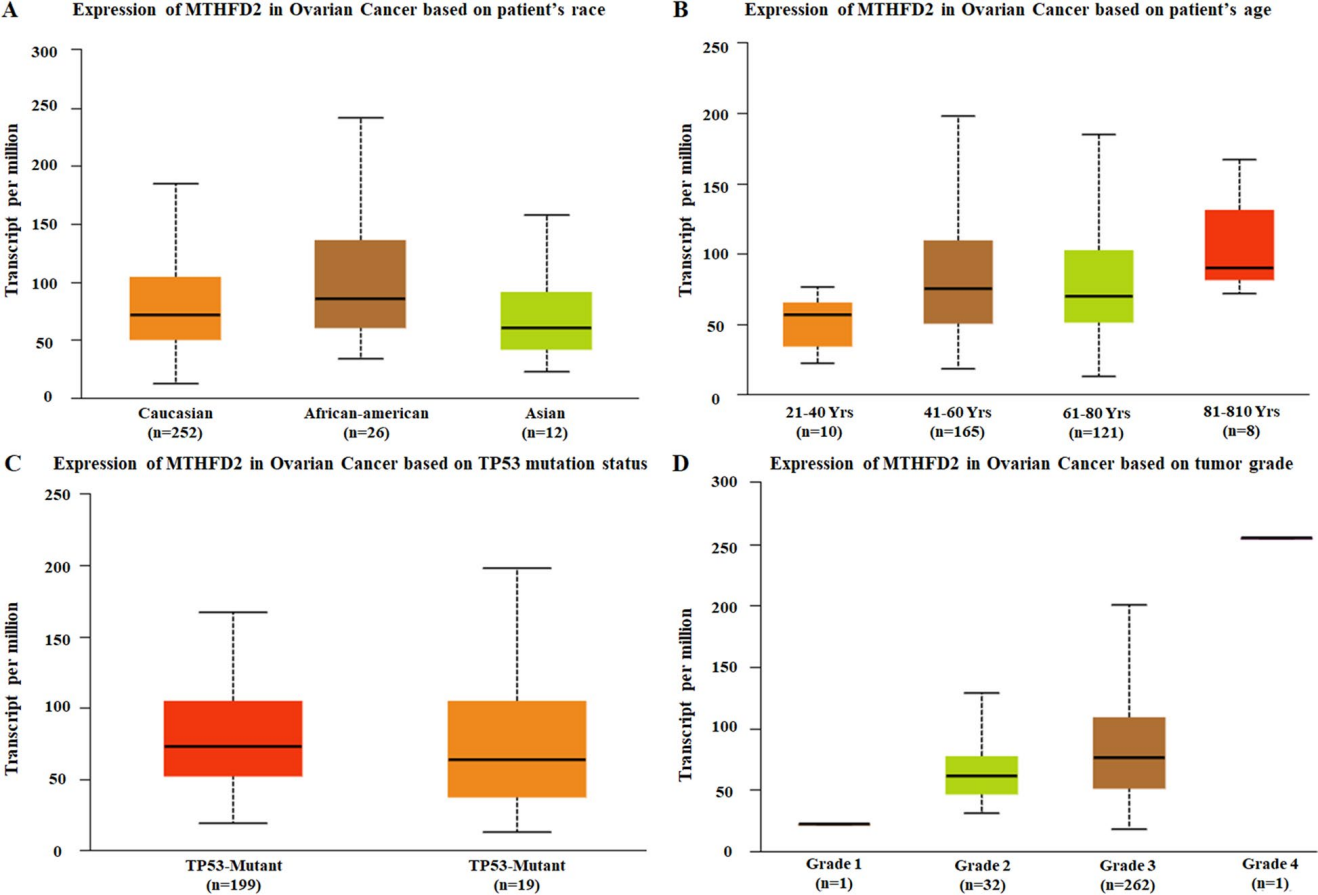
Genetic alterations of MTHFD2 in ovarian cancer subgroups. Subgroup analysis of multiple clinic pathologic features of ovarian cancer samples in the TCGA. **A** rases; **B** Ages; **C** TP53-Mutant; **D** stages

The expression profile of MTHFD2 was identified by GEPIA. The data revealed that MTHFD2 was significantly higher in COAD, DLBC, ESCA, GBM, HNSC, LGG, LUSC, OV, READ, STAD, TGCT, THYM and lower in LAML, THCA (Fig. 1A and B). MTHFD2 transcripts were elevated significantly in ovarian cancer samples compared to normal tissue (*P* < 0.05) **(**Fig. 1C). To further confirmed this result, the Oncomine database was performed to analyze the expression profile of MTHFD2. Elevated mRNA expression of MTHFD2 was identified in various human tumors, including Brain and CNS Cancer, Breast Cancer, Cervical Cancer, Colorectal Cancer, Esophageal Cancer, Head and Neck Cancer, Kidndy Cancer, Leukemia, Liver Cancer, Lung Cancer, Lymphoma, Ovarian Cancer, Prostate Cancer, and Sarcoma (Fig. 2A). MTHFD2 expression was significantly elevated in ovarian mucinous adenocarcinoma, ovarian serous adenocarcinoma, ovarian endometrioid adenocarcinoma than in normal samples (Fig. 2B). Further subgroup analysis according multiple clinical and pathological features of ovarian carcinoma based on TCGA OV samples using UALCAN was revealed in Fig. 3. The expression level of MTHFD2 was positively associated with age and tumor grade.

### MTHFD2 mutations in ovarian cancer

**Fig. 4:**
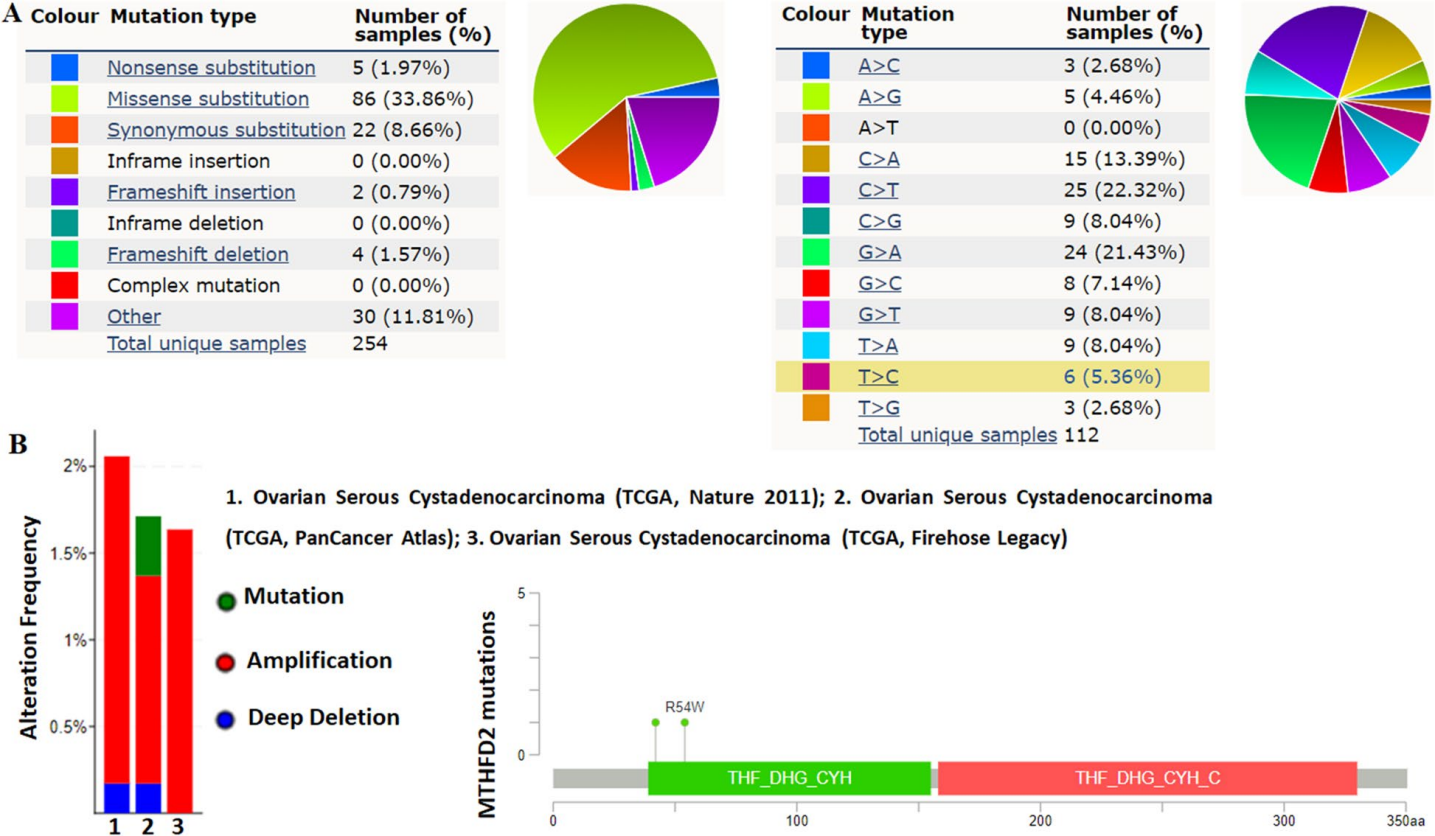
MTHFD2 mutations in human ovarian cancer. **A** The pie chart generated by COSMIC summarizes the observed mutation types, including nonsense substitutions, missense substitutions, synonymous substitutions, inframe insertions, frameshift insertions, inframe deletions, frameshift deletions, and complex mutations. **B** As determined using cBioPortal, the MTHFD2 mutation frequency (0.34%) in patients with ovarian cancer

The pie chart in Fig. 4A generated by COSMIC summarizes the observed mutation types, including nonsense substitutions (1.97%), missense substitutions (33.86%), synonymous substitutions (8.66%), in-frame insertions (0.00%), frameshift insertions (0.79%), inframe deletions (0.00%), frameshift deletions (1.57%), and complex mutations (0.00%). Furthermore, MTHFD2 mutations in ovarian cancer samples were A > C (2.68%), A > G (4.46%), A > T (0.00%), C > A (13.39%), C > T (22.32), C > G (8.04), G > A (21.43%), G > C (7.14%), G > T (8.04), T > A (8.04%), T > C (5.36%), T > G (2.68%). As determined using cBioPortal, the MTHFD2 mutation frequency (0.34%) in patients with ovarian cancer. Furthermore, the additional information about all mutations (G42V and R54W) in MTHFD2 was revealed in graphical view.

### Relationship between MTHFD2 expression and infiltrating immune cells in ovarian cancer

**Fig. 5:**
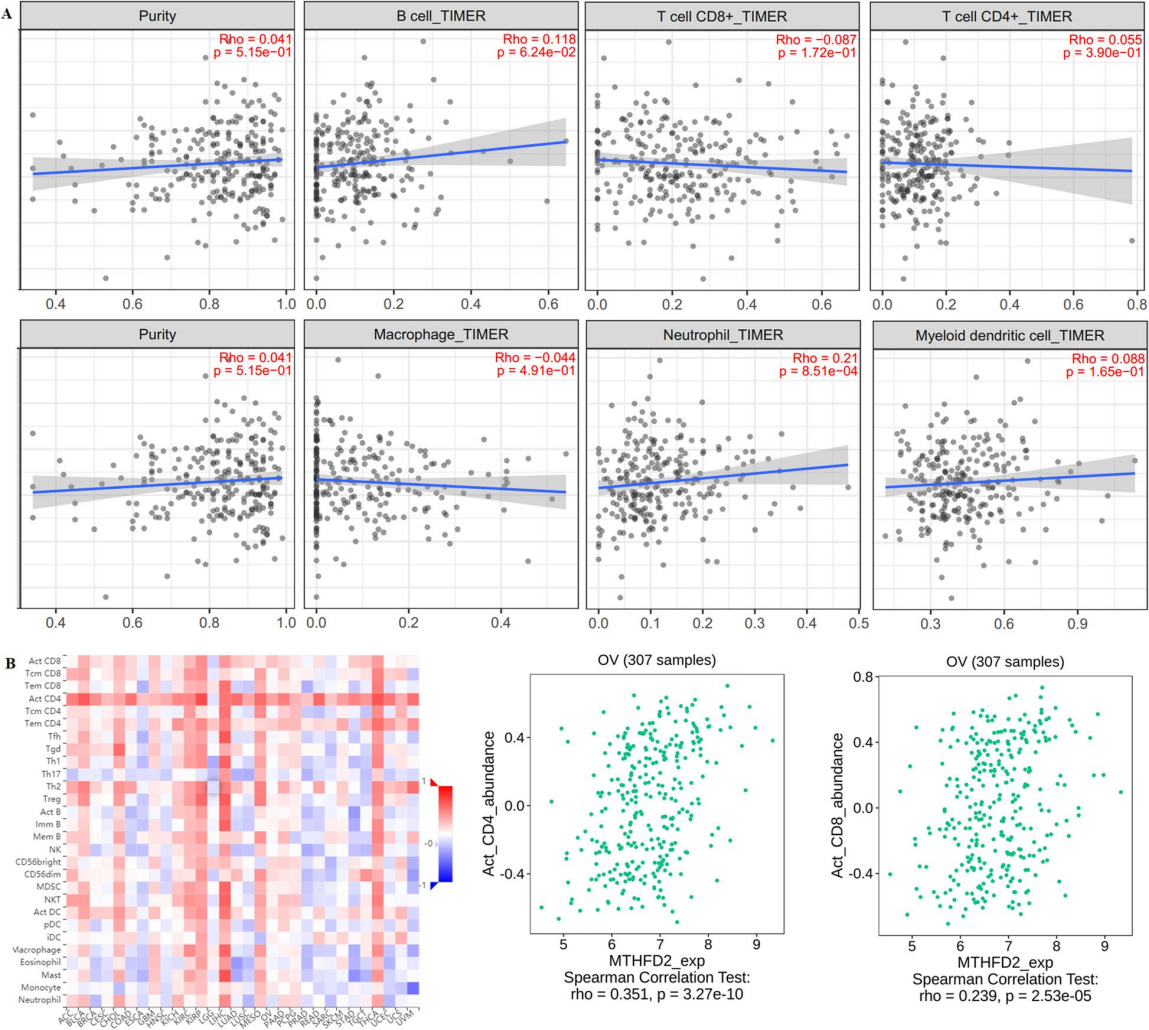
Correlation analysis of MTHFD2 level an immune cells infiltration levels across human cancers using the TIMER dataset and TISIDB database. **A** MTHFD2 exrpession is only significantly positively correlated with neutrophils in ovarian cancer. **B** MTHFD2 is related with 7 immune cell subtypes in ovarian cancer

Tumor-infiltrating immune cells in the TME can independently be adopt to predict sentinel lymph node status and patient survival, and the above findings support a prognostic role of MTHFD2 in pan-cancer [4, 24]. Therefore, we used TIMER to explore the association between immune infiltration levels and MTHFD2 expression level in various cancer types. The results showed that MTHFD2 exrpession is only significantly positively correlated with neutrophils (r = 0.21, *p* = 8.51 × 10^− 4^) in ovarian cancer (Fig. 5A). Subsequently, we used TISIDB database to further explore the association between MTHFD2 expression level and 28 tumor immune infiltrating cell subtypes. These results revealed that MTHFD2 is related with 7 immune cell subtypes in ovarian cancer (Fig. 5B**,** Supplement 1, and Table 1). Activated CD8 T cell (r = 0.239, *p <* 2.53e-05), activated CD4 T cell (r = 0.351, *p <* 3.27e-10), effector memory CD4 T cell (r = 0.186, *p <* 0.00105), CD56 bright natural killer cell (r = 0.151, *p <* 0.00831), CD56 dim natural killer cell (r = 0.137, *p <* 0.016), activated dendtritic cell (r = 0.118, *p <* 0.0384), neutrophil (r = − 0.114, *p <* 0.0468) are weakly correlated with MTHFD2. These results implicate that MTHFD2 plays a weak role in tumor immune infiltration regulator of ovarian cancer.

**Table 1:** The correlation between MTHFD2 expression and tumor lymphocyte infiltration in ovarian cancer (TISIDB)

Tumor lymphocyte infiltration	r	***P***
Activated CD8 T cell (Act_CD8)	0.239	**2.53e-05**
Central memory CD8 T cell (Tcm_CD8)	0.025	0.664
Effector memory CD8 T cell (Tem_CD8)	0.039	0.492
Activated CD4 T cell (Act_CD4)	0.351	**3.27e-10**
Central memory CD4 T cell (Tcm_CD4)	−0.226	0.654
Effector memory CD4 T cell (Tem_CD4)	0.186	**0.00105**
T follicular helper cell (Tfh)	−0.033	0.564
Gamma delta T cell (Tgd)	0.05	0.386
Type 1 T helper cell (Th1)	0.004	0.94
Type 17 helper cell (Th17)	−0.039	0.491
Type 2 helper cell (Th2)	−0.014	0.811
Regulatory T cell (Treg)	0.058	0.309
Activated B cell (Act_B)	0.033	0.564
Immature B cell (Imm_B)	0.043	0.448
Memory B cell (Mem_B)	−0.022	0.696
Natural killer cell (NK)	−0.012	0.838
CD56 bright natural killer cell (CD56 bright)	0.151	**0.00831**
CD56 dim natural killer cell (CD56 dim)	0.137	**0.016**
Myeloid derived suppressor cell (MDSC)	0.057	0.322
Natural killer T cell (NKT)	0.051	0.374
Activated dendtritic cell (Act_DC)	0.118	**0.0384**
Plasmacytoid dendtritic cell (pDC)	−0.001	0.981
Immature dendtritic cell (iDC)	0.059	0.3
Macrophage (Macrophage)	−0.025	0.669
Eosinophi (Eosinophil)	−0.044	0.438
Mast (Mast)	0.016	0.775
Monocyte (Monocyte)	0.095	0.0955
Neutrophil (Neutrophil)	−0.114	**0.0468**

### Relationship of MTHFD2 expression and prognosis in ovarian cancer

**Fig. 6:**
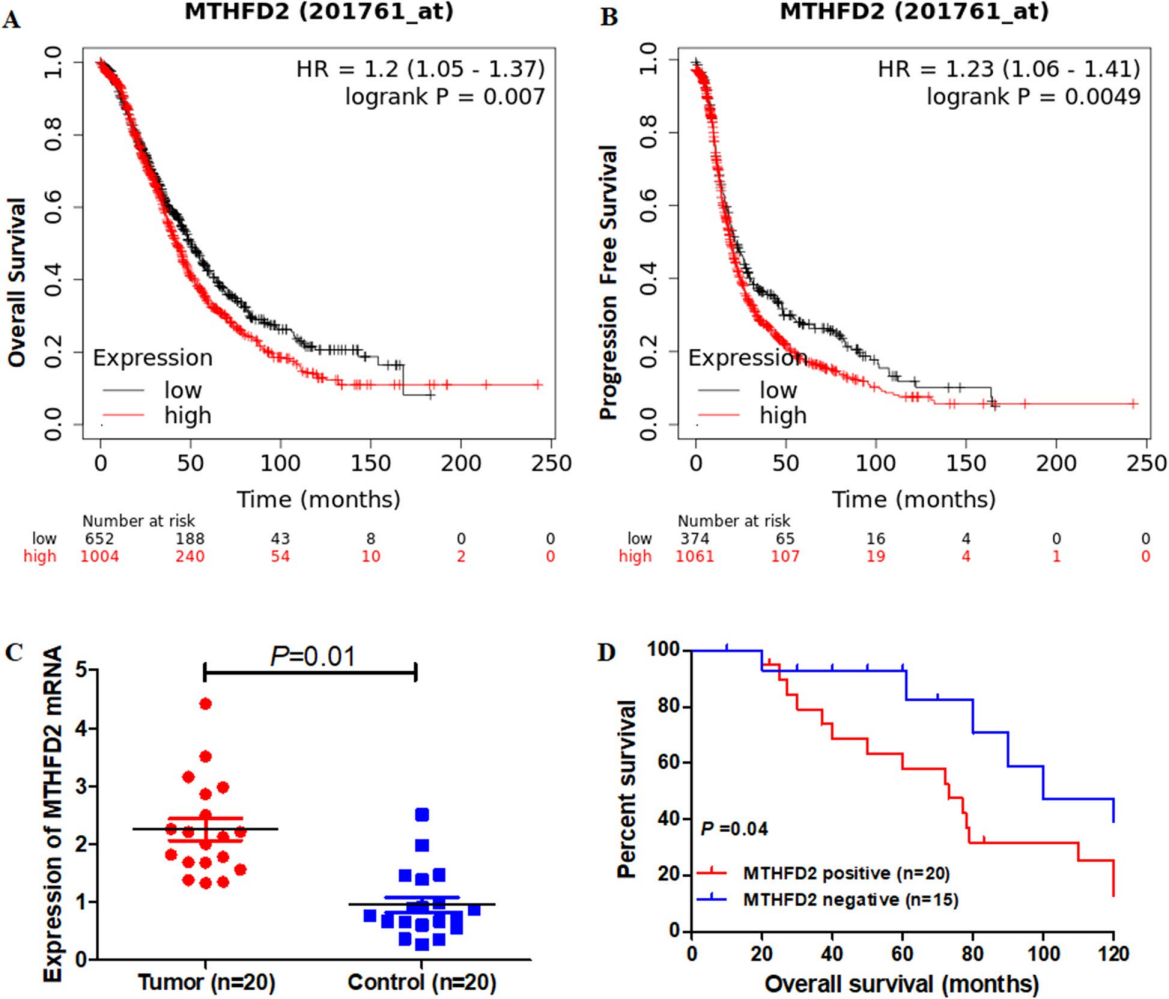
MTHFD2 as a prognosis marker in ovarian cancer. **A** Overall survival (OS) curves calculated by Kaplan-Meier Plotter Analysis. **B** Progression Free Survival (PFS) curves calculated by Kaplan-Meier Plotter Analysis. **C** Expression of MTHFD2 in tumor (20 cases) and adjacent normal tissues (20 cases). **D** Kaplan-Meier curves based on MTHFD2 expression were drawn for overall survival in 35 patients

To evaluate whether MTHFD2 expression level has predictive significance for ovarian carcinoma prognosis, we detected the expression of MTHFD2 mRNA and its relationship with prognosis. As shown in Fig. 6A and B, the MTHFD2 expression was negatively related to overall survival (HR = 1.2 [1.05–1.37], *p* = 0.007) and progression free survival (HR = 1.23 [1.06–1.41], *p* = 0.0049). Furthermore, we detected our own clinical samples found that the overexpression of MTHFD2 mRNA and worse probabilities of survival in ovarian cancer (Fig. 6C and D).

### Co-expression and enrichment analysis of MTHFD2 in ovarian cancer

**Fig. 7:**
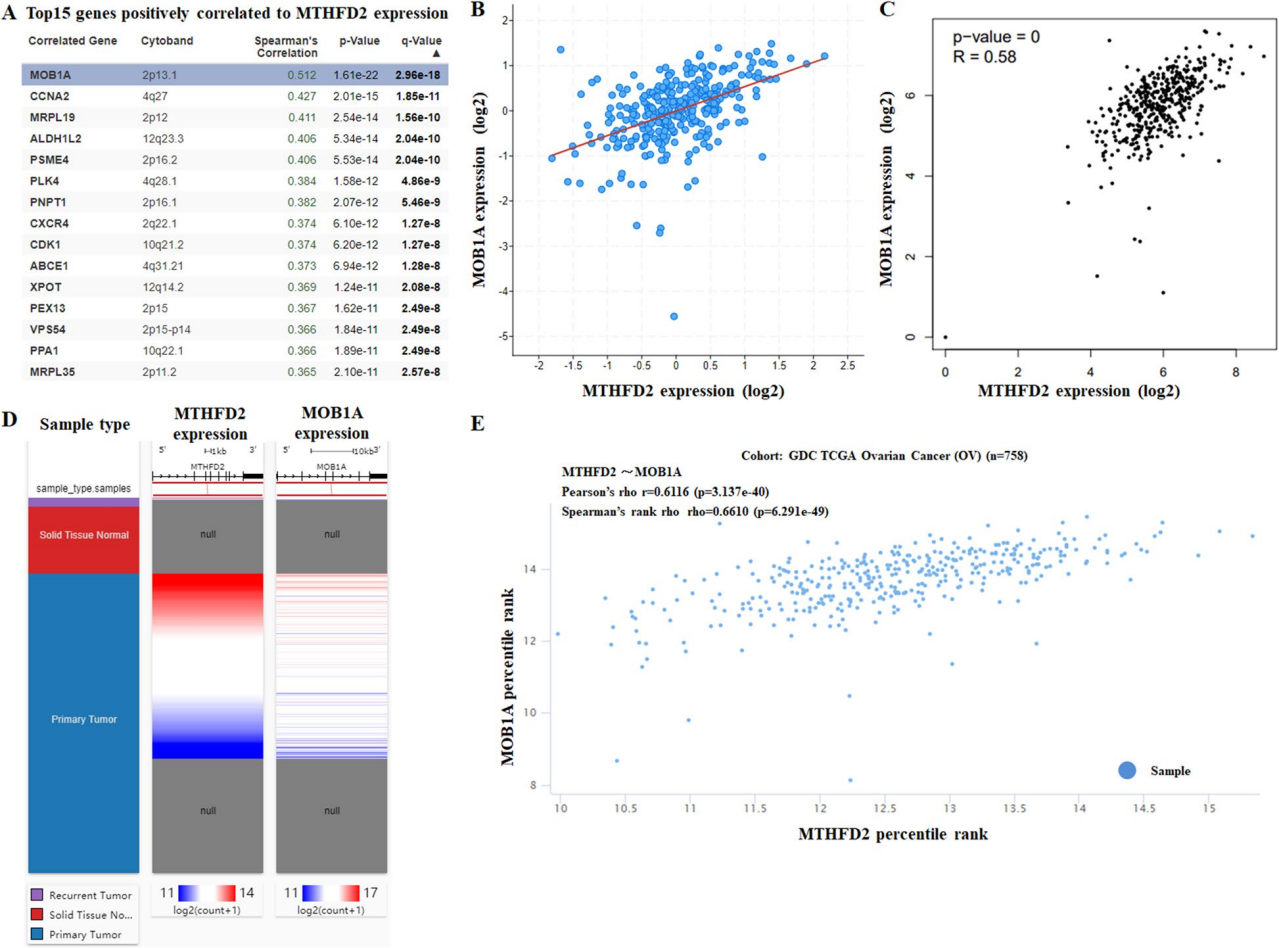
Co-Expression of MTHFD2 gene. **A** Co-expression of MTHFD2 gene as detected by cBioPortal. **B** Regression analysis between MTHFD2 and MOB1A in ovarian cancer determined by cBioPortal. **C** Relationship between MTHFD2 and MOB1A in ovarian cancer by GEPIA. **D** Heat map of MTHFD2 and MOB1A mRNA expression in ovarian cancer identified by UCSC Xena. **E** Correlation between MTHFD2 and MOB1A mRNA expression in the TCGA database, identified by UCSC Xena

**Fig. 8:**
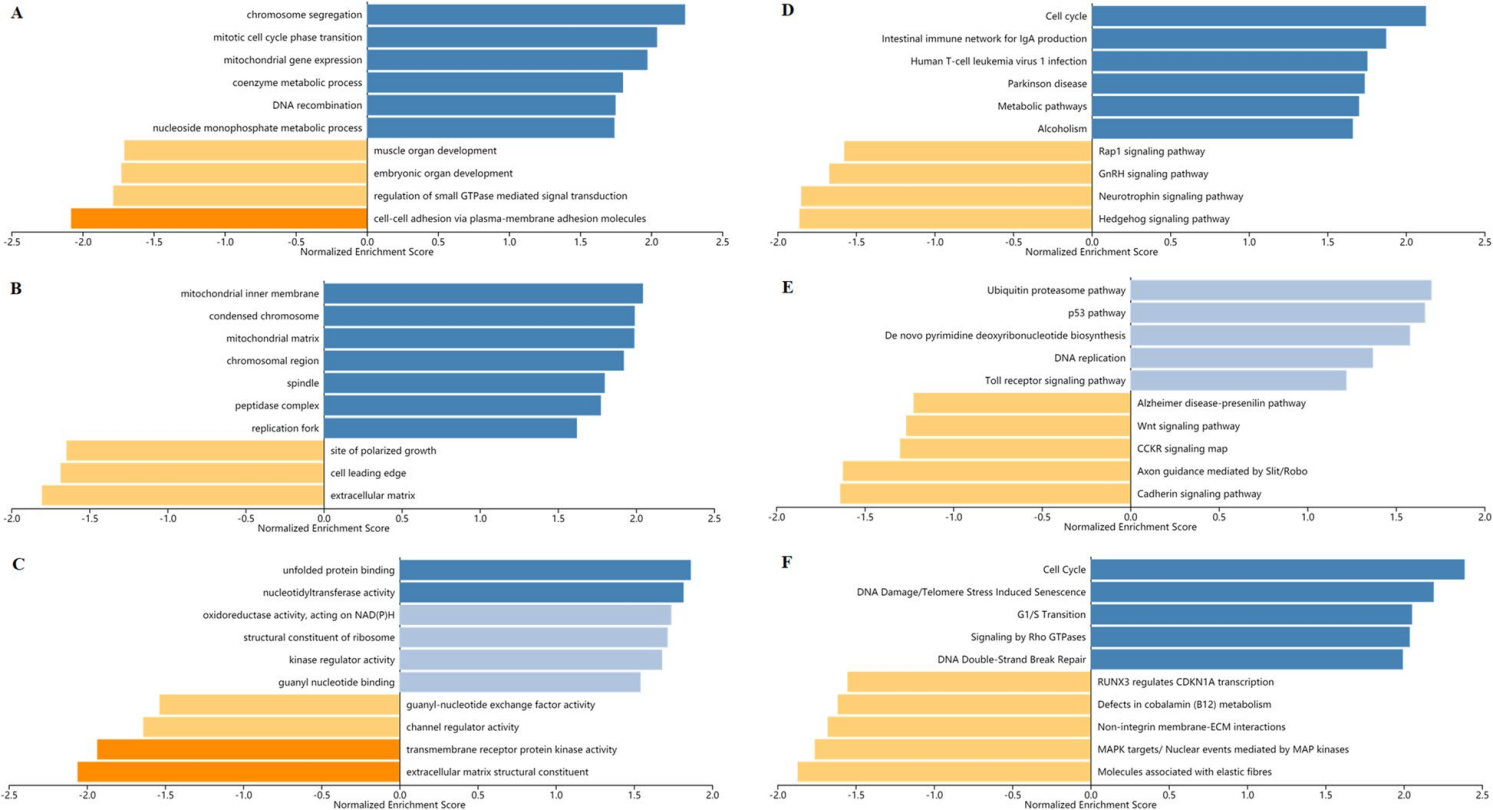
Enrichment analysis of MTHFD2 functional networks in ovarian cancer. **A** Biological process. **B** Cellular component. **C** Molecular function. **D** KEGG pathway. **E** PANTHER pathway. **F** Reactome pathway

To further evaluate the regulatory mechanisms underlying the role of MTHFD2 in ovarian cancer, data mining was performed on a ovarian cancer cohort using cBioPortal. Mps one binder kinase activator 1A (MOB1A) is a highly related gene (Fig. 7A), which involves in the regulation of cell survival, proliferation, differentiation and organogenesis [32]. A regression analysis using cBioportal demonstrated that *MTHFD2* and *MOB1A* levels are highly correlated (Spearman’s correlation = 0.512, *p*-value = 1.61e-22) (Fig. 7B). The positive correction between *MTHFD2* and *MOB1A* mRNA expression was determined using data from GEPIA (Fig. 7C). By investigating ovarian cancer data in TCGA using UCSC Xena, the positive correlation was further confirmed (Pearson’s correlation = 0.6116; Spearman’s correlation = 0.6610) (Fig. 7D and E). These data informed that *MTHFD2* could be associated with the *MOB1A* pathway in ovarian cancer. Furthermore, three independent ontologies (biological process, cellular component, and molecular function) were analyzed by gene set enrichment analysis (GSEA). The results indicated that MTHFD2 mainly involved in several biological processes (chromosome segregation, mitotic cell cycle phase transition, mitochondrial gene expression, etc.) (Fig. 8A), cellular components (mitochondrial inner membrane, condensed chromosome, mitochondrial matrix, etc.) (Fig. 8B), and molecular functions (unfolded protein binding, nucleotidytransferase activity, oxidoreductase activity, acting on NAD(P) H, etc.) (Fig. 8C). Then we evaluated the potential functional pathways using KEGG (cell cycle, intestinal immune network for IgA production, human T-cell leukemia virus 1 infection, etc.) (Fig. 8D), PANTHER (ubiquitin proteasome pathway, p53 pathway, de novo pyrimidine deoxyribonucleotide biosynthesis, etc.) (Fig. 8E), and Reactome (cell cycle, DNA damage/telomere stress induced senescence, G1/S transition, etc.) (Fig. 8F).

### MOB1A mRNA expression and prognosis in patients with ovarian cancer

**Fig. 9:**
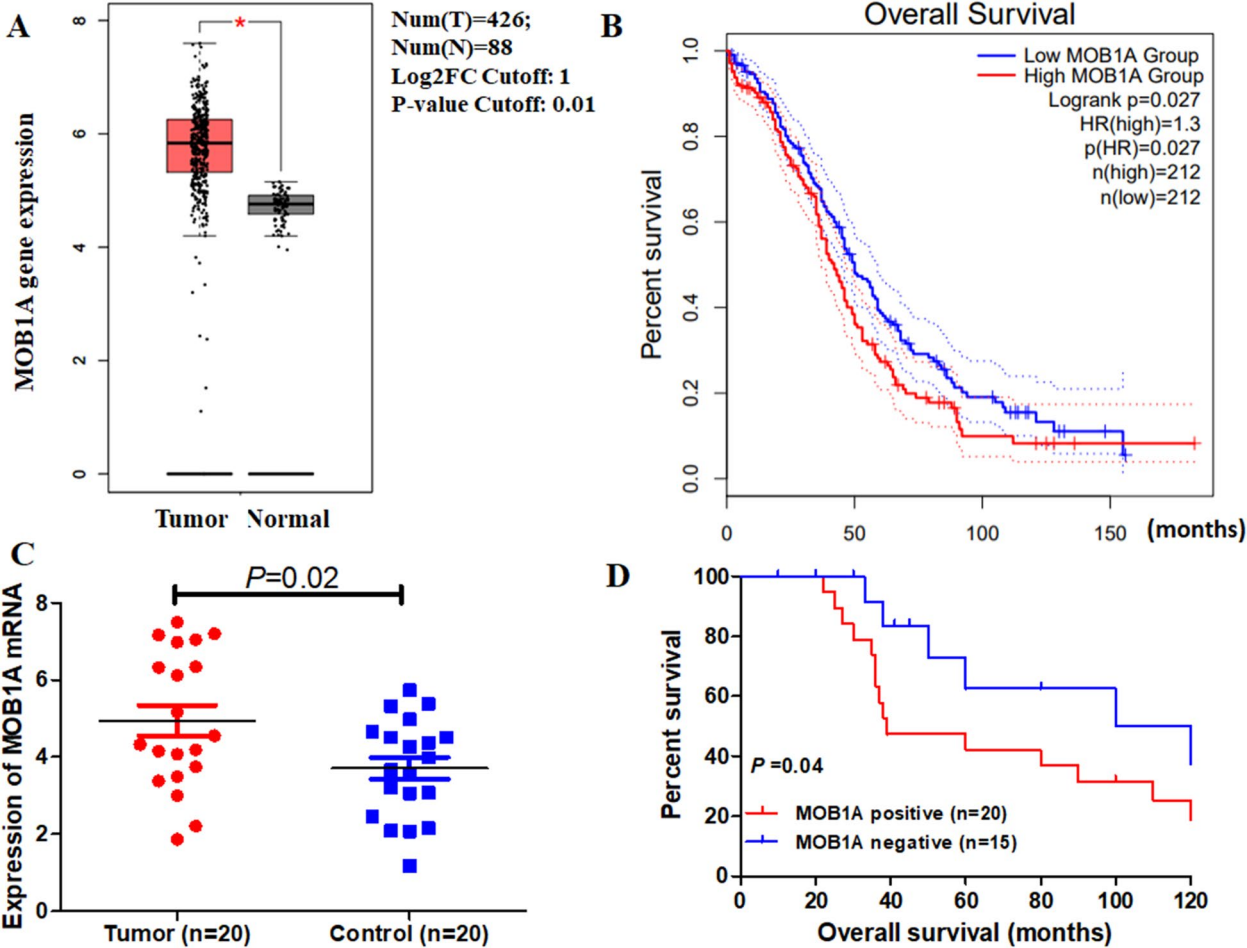
Expression of MTHFD2 in ovarian cancer. **A** The expression of MOB1A mRNA in ovarian cancer tissues (red box) and paired normal tissues (black box) from GEPIA. **B** Overall survival (OS) curves calculated by PrognoScan database. **C** Expression of MOB1A in tumor (20 cases) and adjacent normal tissues (20 cases). **D** Kaplan-Meier curves based on MOB1A expression were drawn for overall survival in 35 patients

To investigate the genetic alterations of MOB1A, the GEPIA tool was performed to identify the expression profiles of MOB1A. The results of MOB1A analysis informed that MOB1A highly expressed in ovarian cancer tissues compared with matched normal tissues (Fig. 9A). Then, the prognostic value of MOB1A in ovarian cancer was identified by PrognoScan database. It was demonstrated that MOB1A mRNA expression was significantly associated with reduced overall survival (OS) time in ovarian cancer (Fig. 9B). Furthermore, our own results demonstrated the over-expression of MOB1A mRNA and worse probabilities of survival in ovarian cancer (Fig. 9C and D).

### MTHFD2 accelerated the biological characteristics of ovarian cancer cells through up-regulating MOB1A

**Fig. 10:**
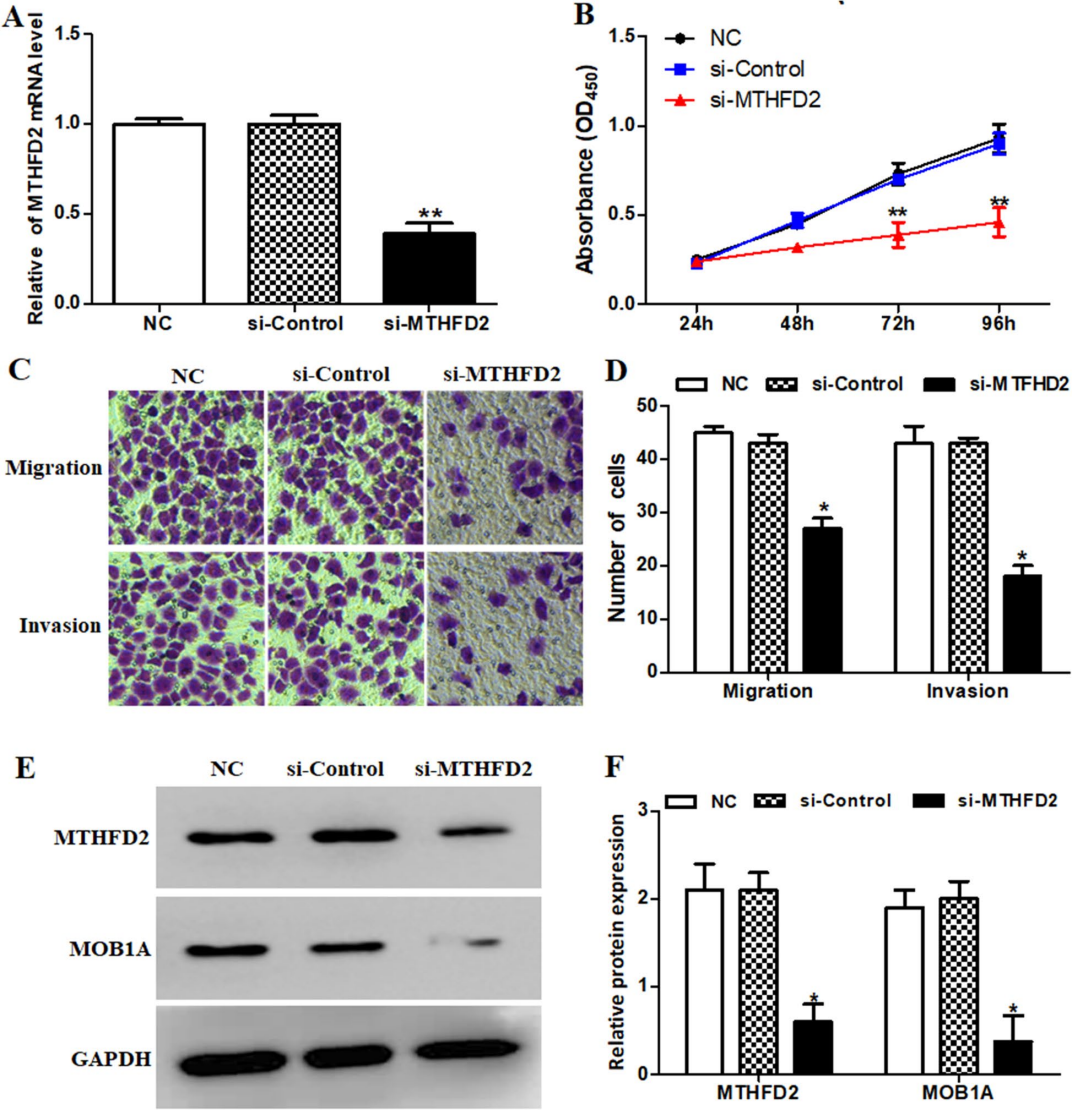
MTHFD2 accelerated the cell growth, invasion, and migration in ovarian cancer through MOB1A. **A** The expression of MTHFD2 was significantly reduced in ovarian cancer cells transfected with si-MTHFD2. **B** The cell proliferation of ovarian cancer cell lines transfected with different interference sequences, cell proliferation was the weakest in si-MTHFD2 group. **C** and **D** Invasion and migration assays of ovarian cancer cells under different interference conditions. **E** and **F** The western blot revealed MOB1A expression was significantly decreased in si-MTHFD2 group ovarian cells. *P < 0.05 and **P < 0.01, compared with NC group

To further confirm the impact of MTHFD2 on ovarian cancer tumorigenesis, the endogenous expression level of MTHFD2 was knocked down with si-MTHFD2 transfection. As exhibited in Fig. 10A, the MTHFD2 expression was significantly reduced in ovarian cancer cells transfected with si-MTHFD2. Then, CCK-8 assay demonstrated that MTHFD2 knock-down significantly weakened the ability of proliferation of ovarian cancer (Fig. 10B). Furthermore, the transwell assay showed that knockdown of MTHFD2 obviously limited cell migration and invasion (Fig. 10C and D). Finally, the expression of MOB1A was inhibited by MTHFD2 in ovarian cancer cells (Fig. 10E and F). Above data elucidated that MTHFD2 could promot ovarian cacncer cell progression by up-regulating MOB1A in vitro.

## Discussion

MTHFD2 is a NAD^+^-dependent enzyme with methylenetetrahydrofolate dehydrogenase and cyclohydrolase activity, which has been suggested to be a key participant in the metabolic reprogramming and tumor cell-sustaining proliferative capacity independently of dehydrogenase activity [10, 16]. Increasing evidence suggests that MTHFD2 is overexpressed and predicts poor prognosis and promotes cancer cell proliferation and metastasis in certain types of malignancies, including colorectal cancer [29], breast cancer [8], and head and neck squamous cell cancer [8, 25]. However, the prognostic value and signal transduction of MTHFD2 expression in ovarian cancer is still ambiguous.

In the present study, to determine the role and functional network of MTHFD2 in the ovarian cancer, we performed bioinformatics analyses to analyze the extensive gene expression profiling with pre-defined parameters in ovarian cancer and normal samples. According to GEPIA database, MTHFD2 expression was significantly increased in ovarian cancer tissues compared with adjacent normal controls. The analysis of the Oncomine database revealed fold-changes in gene expression levels that MTHFD2 expression was significantly increased in ovarian mucinous adenocarcinoma, ovarian serous adenocarcinoma, ovarian endometrioid adenocarcinoma compared with normal samples. Further subgroup analysis according multiple clinical and pathological features of ovarian carcinoma based on TCGA OV samples using UALCAN. The expression level of MTHFD2 was positively associated with age and tumor grade. Subsequently, the mutations of MTHFD2 were identified by COSMIC and cBioPortal database. We found that the main type of mutation of MTHFD2 in ovarian cancer was missense substitutions mainly occurred in THF_DHG_CYH domain. Then, the relationship between MTHFD2 expression and infiltrating immune cells was identified by TIMER and TISIDB databse. These results suggested that MTHFD2 plays a weak role in tumor immune infiltration regulator of ovarian cancer. Prognosis analysis showed that the higher expression of MTHFD2 positively correlated with reduced OS and PFS. We further adopt our own results validated that that MTHFD2 mRNA expression may be an independent prognostic biomarker in ovarian cancer patients. Enrichment analysis of target gene sets using GSEA can identify important networks involved in protein targeting, mRNA processing, and transcription factors. MTHFD2 was showed to regulate the ubiquitin proteasome pathway, p53 pathway, de novo pyrimidine deoxyribonucleotide biosynthesis.

By mining co-expression and correlation analysis data, we determined that MTHFD2 and MOB1A were both upregulated in ovarian cancer. Mps One Binder Kinase Activator (MOB)1A, one of the most core components of the Hippo pathway, was considered to influence biological functions in various cancers [17, 18, 31]. Furthermore, multiple studies have revealed that MOB1A is involved in development and progress in many types of cancers, such as glioma [30], gallbladder carcinoma [32], cervical carcinoma [19], pancreatic ductal adenocarcinoma [21], and gastric cancer [31]. Then, we identified the expression of MOB1A in ovarian cancer through GEPIA database and our own samples. The results demonstrated that MOB1A mRNA expression was significantly up-regulated and correlated with poor survival in ovarian carcinoma samples compared with controls. These results illustrated that the expression of MOB1A may regulate tumor invasion and survival correlated with MOB1A.

Subsequently, we further identified the function of MTHFD2 in growth and metastasis of ovarian cancer cells through knockdown the expression of MTHFD2 using siRNA technology. Our experiments confirmed that MTHFD2 might be a potential factor promoting the proliferation, migration, and invasion of ovarian cancer. Consistently, after knock down MTHFD2, we found that the expression of MOB1A was significantly decreased in ovarian cancer cell lines. These experiments preliminarily demonstrate that MTHFD2 exerted its effects on the malignant behaviors of ovarian cancer cells through the MOB1A signaling pathway.

## Conclusion

In summary, through our study found that MTHFD2 is highly expressed in ovarian cancer, as well as an indispensable risk factor for poor prognosis in patients with ovarian cancer. We also found that that MTHFD2 has a role in malignancy mainly through the MOB1A signaling, which is a potential target for treating ovarian cancer. However, some limitations remain as the potential regulatory mechanism of MTHFD2 in ovarian were not verified sufficiently and required further exploration.

## Supplementary Information

**Additional file 1.**

## Data Availability

The analyzed data sets generated during the study are available from the corresponding author on reasonable request.
