# Supplementary figures and images for: Up-regulation of MTHFD2 is associated with clinicopathological characteristics and poor survival in ovarian cancer, possibly by regulating MOB1A signaling
Source: J Ovarian Res. 2022 Feb 8;15:23. doi: 10.1186/s13048-022-00954-w (PMC8827288; doi:10.1186/s13048-022-00954-w)

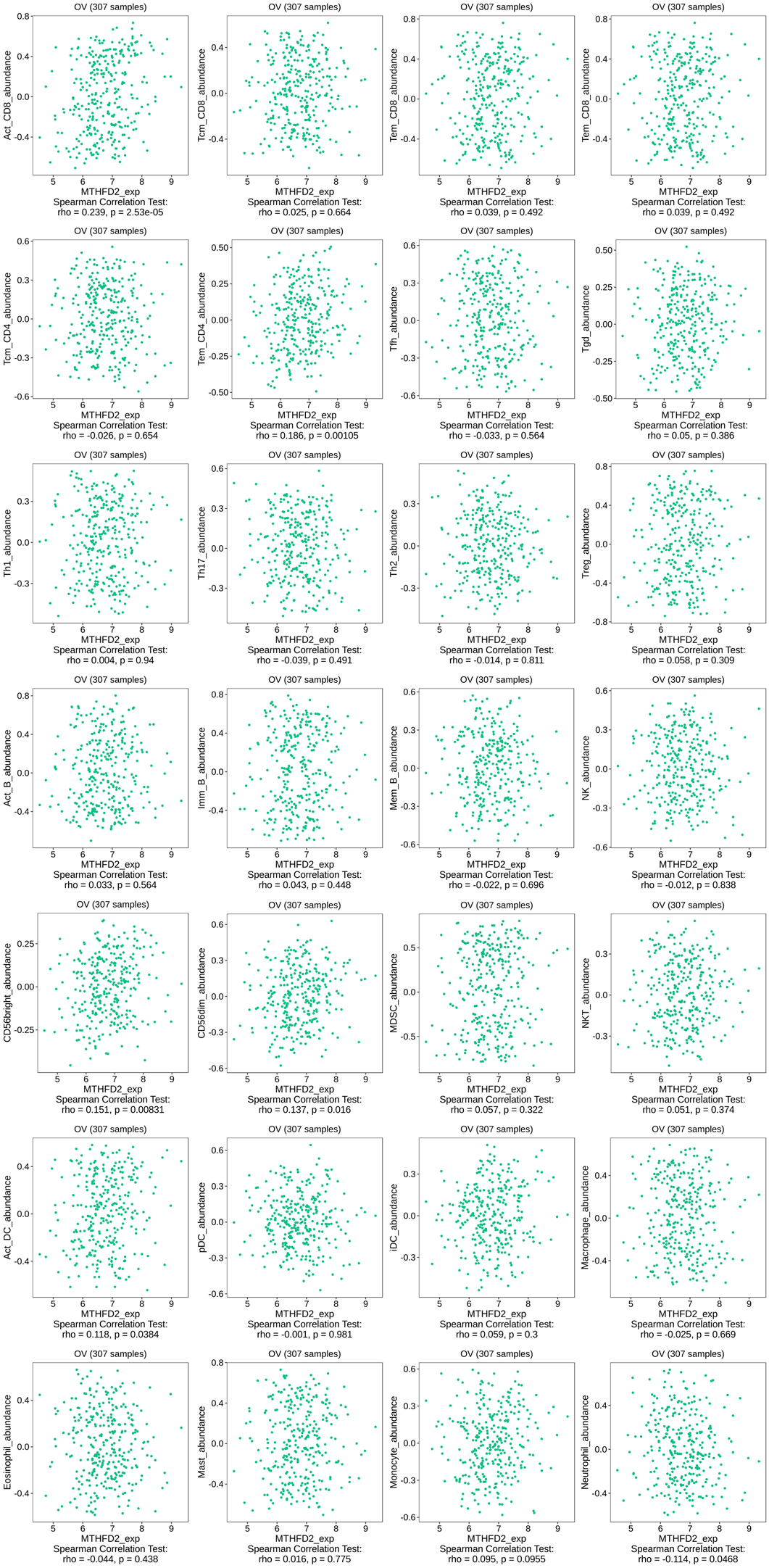

Supplement: Supplementary file 1 — Additional file 1. [file 13048_2022_954_MOESM1_ESM.tif]
